# Concurrence in the ability for lipid synthesis between life stages in insects

**DOI:** 10.1098/rsos.160815

**Published:** 2017-03-22

**Authors:** Bertanne Visser, Denis S. Willett, Jeffrey A. Harvey, Hans T. Alborn

**Affiliations:** 1 Evolutionary Ecology and Genetics Group, Biodiversity Research Centre, Earth and Life Institute, Université Catholique de Louvain, Croix du Sud 4-5, 1348 Louvain-la-Neuve, Belgium; 2 Institut de Recherche sur la Biologie de l’Insecte (IRBI), UMR 7261 CNRS/Université François-Rabelais de Tours, Avenue Monge, 37200 Tours, France; 3 Chemistry Research Unit, Center of Medical, Agricultural, and Veterinary Entomology, Agricultural Research Service, United States Department of Agriculture, 1600 SW 23rd Drive, Gainesville, FL 32608, USA; 4 Department of Ecological Sciences, VU University Amsterdam, Section Animal Ecology, De Boelelaan 1085, 1081HV Amsterdam, The Netherlands; 5 Department of Terrestrial Ecology, Netherlands Institute of Ecology, Droevendaalsesteeg 10, 6700 EH Wageningen, The Netherlands

**Keywords:** fatty acid synthesis, metabolism, deuterium, evolution

## Abstract

The ability to synthesize lipids is critical for an organism’s fitness; hence, metabolic pathways, underlying lipid synthesis, tend to be highly conserved. Surprisingly, the majority of parasitoids deviate from this general metabolic model by lacking the ability to convert sugars and other carbohydrates into lipids. These insects spend the first part of their life feeding and developing in or on an arthropod host, during which they can carry over a substantial amount of lipid reserves. While many parasitoid species have been tested for lipogenic ability at the adult life stage, it has remained unclear whether parasitoid larvae can synthesize lipids. Here we investigate whether or not several insects can synthesize lipids during the larval stage using three ectoparasitic wasps (developing on the outside of the host) and the vinegar fly *Drosophila melanogaster* that differ in lipogenic ability in the adult life stage. Using feeding experiments and stable isotope tracing with gas chromatography/mass spectrometry, we first confirm lipogenic abilities in the adult life stage. Using topical application of stable isotopes in developing larvae, we then provide clear evidence of concurrence in lipogenic ability between larval and adult life stages in all species tested.

## 1. Introduction

The ability to synthesize lipids is critical for an organism’s fitness because it provides the energetic and structural resources necessary to sustain life. For example, to prepare for diapause (a time at which no food will be available), developing insects will accumulate a greater amount of lipid reserves [1]. Reproductive investment also relies heavily on lipids, as lipids comprise a major part of the macronutrient content allocated towards eggs in birds and insects [2–7]. Lipids are further an essential component of cell membranes, whose composition determines membrane fluidity, an important process during temperature acclimation [8]. Lipid synthesis is indeed highly conserved across organisms which will readily and involuntarily convert sugars and other carbohydrates into lipids once immediate nutritional demands have been met [9,10]. When a surplus of carbohydrates is available, pyruvate generated through glycolysis will be used to produce acetyl coenzyme A (acetyl-CoA) [11,12], a central intermediate for nutrient metabolism. Acetyl-CoA is then carboxylated to form malonyl-CoA, which is used for the formation of fatty acids (FAs) with a chain length of 16 carbon atoms, i.e. palmitate (C16:0). Palmitate can then be elongated to form longer-chain FAs, desaturated to generate unsaturated FAs or used for the synthesis of storage lipids, i.e. triglycerides [13].

The ability for lipid synthesis was long thought to be ubiquitous among animals. It was thus surprising that during the 1990s and 2000s several researchers independently uncovered that some parasitic wasp species were not capable of synthesizing lipids [14]. Most of these studies either used gravimetry (i.e. based on measuring weights; [15,16]) or colourimetry (i.e. based on measuring light absorbance; [17,18]) to show that lipid levels decreased throughout a wasp’s life despite superfluous access to a sugar source. While these methods can reveal decreasing lipid levels, they cannot be used to exclude the possibility that lipids are burned at a faster rate than at which they are produced, i.e. stable lipids loss [19]. Using a liquid scintillator analyser (i.e. which measures levels of ionizing radiation), Giron & Casas [20] showed that radioactively labelled glucose was not incorporated into the lipid fraction of their model parasitic wasp species. This study thus refuted the hypothesis that decreasing lipid levels were the result of stable lipid loss in this species. Similar results using chromatography-based methods later confirmed these findings in another wasp [21].

A comparative study revealed that the lack of lipid synthesis predominates among adult insects adopting a parasitoid lifestyle (including parasitoid wasps, beetles and flies), with only a few exceptions of parasitoid species that synthesize lipids [22]. This study indeed revealed that lipid synthesis re-evolved only in a few species with a broad host range, i.e. generalists. The parasitoid lifestyle, in which insects develop in or on an arthropod host, thus allows the larval stages of mainly specialist parasitoids to carry over substantial lipid reserves from the host [23] rather than from synthesizing lipids de novo. While many parasitic wasp species have by now been tested for their capacity to synthesize lipids in the adult life stage [14,22], so far only two studies have indirectly addressed parasitoid lipid synthesis at the larval stage with conflicting results. In both cases, parasitoid larvae were reared on a lipid-free artificial medium, in which one of the species was able to survive into adulthood, whereas the other species was not [24,25]. It has remained unclear, however, whether these species are able to synthesize lipids in the adult life stage. Ideally, the ability for lipid synthesis in parasitoid larvae should be tested under more natural conditions, such as in association with their natural host. The intricate relationship between host and parasitoid makes it difficult, however, to tease apart metabolic functions of host versus parasitoid, particularly in endoparasitoids that live inside their host’s body.

It has thus far remained unclear whether (the lack of) lipid synthesis is concurrent between life stages. Here we determined the ability for lipid synthesis in several insects during both the larval and adult stages. Experiments were performed on three ectoparasitic wasps (developing on the outside of the host), as well as the vinegar fly *Drosophila melanogaster*, which differ in lipogenic ability in the adult life stage. If the ability for lipid synthesis was lost and regained in wasp species that lack or synthesize lipids (as previously suggested [22]), we would expect similar lipogenic abilities throughout the life stages. Alternatively, a threshold model could apply where the quantity of lipids obtained from the host during development determines whether additional lipids should be synthesized during adult life, i.e. lipid synthesis is plastic. In the latter case, we would expect larvae of all species to synthesize lipids (albeit at a lower rate when the host contains high lipid levels). Using feeding experiments and stable isotope tracing with gas chromatography/mass spectrometry (GC/MS), we first confirm lipogenic abilities in the adult life stage. Using topical application of stable isotopes in developing larvae, we then provide clear evidence of concurrence in lipogenic ability between larval and adult life stages in all species tested.

## 2. Material and methods

### 2.1. Insects

***Drosophila melanogaster*** (Diptera: Drosophilidae). Individuals were obtained from a culture that was originally collected in Dwingeloo, The Netherlands (latitude: +52°50′39.12′′ N; longitude: +6°23′57.48′′ E) in 2013. Individuals were maintained and experiments performed at a relative humidity of 75%, a photoperiod of 16 L : 8 D, and a temperature of 20°C on a food medium containing agar (20 g l^−1^), sugar (50 g l^−1^), yeast (35 g l^−1^), nipagin (5 ml l^−1^) and propionic acid (5 ml l^−1^). To test larval lipogenic ability, adults from the stock were allowed to oviposit during 6 h on food medium. After 2 days of development (when fat reserves are not yet visible), larvae were removed from flasks and randomly exposed to either approximately 25 nl of isotope solution (*n*=16), containing 0.1% Triton X in deuterated water (i.e. D_2_O; 99.8% atom % D, ACROS Organics) for treated individuals or approximately 25 nl of 0.1% Triton X in water for controls (*n*=15) for about 1 min until the solution was completely absorbed (Triton X is applied to remove the wax layer on the larval cuticle to allow for more efficient absorption of (deuterated) water). Solutions were applied with a pipette, in which each larva was placed in a tiny droplet of solution maximizing absorption through the cuticle. Larvae were then allowed to continue development until 6 days of age, when accumulated fat was clearly visible, and frozen at −18°C until further processing. To test adult lipogenic ability, freshly emerged females were collected from the stock and allowed to feed ad libitum from a 40% sucrose solution in 1 : 1 H_2_O:D_2_O for treated individuals (*n*=12) or a 40% sucrose solution in H_2_O for controls (*n*=10). After 7 days with access to food, adults were frozen at −18°C until further processing.

***Eupelmus vuilleti*** (Hymenoptera: Eupelmidae). *Eupelmus vuilleti* is a solitary ectoparasitoid that lays a single egg on a third to fourth instar *Callosobruchus maculatus* (Coleoptera: Bruchidae) larva. *Eupelmus vuilleti* and its host, *Ca. maculatus* were obtained from an existing culture at the Institute of Research on Insect Biology (Tours University, France). Individuals were maintained and experiments performed at a relative humidity of 65%, a photoperiod of 13 L : 11 D and a temperature regime of 33°C (lights on) : 23°C (lights off). To test larval lipogenic ability, five *Ca. maculatus* host larvae were presented in gelatin capsules (that mimic natural oviposition substrates, [26]) to four *E. vuilleti* stock females per Petri dish (diameter 6 cm). After 24 h, host larvae containing an *E. vuilleti* egg were transferred singly to a small Petri dish (diameter 3 cm). After 3 days of development, each *E. vuilleti* larvae was detached from its host, after which either the isotope (*n*=12) or control solution (*n*=13) was applied randomly in a manner similar to that described for *D. melanogaster*. After complete absorption of the solution, *E. vuilleti* larvae were placed back onto the same host larva to continue development. Once the host was completely consumed, larvae were frozen at −18°C at 7 days of age until further processing. To test adult lipogenic ability, the same procedure was followed as described for *D. melanogaster* (with *n*=13 and *n*=7 for treatment and control groups, respectively).

***Gelis* sp.** *Gelis agilis* and *Gelis areator* (Hymenoptera: Ichneumonidae) are solitary ectoparasitoids that prefer to oviposit on cocoons or cocoon-like structures as hyperparasitoids, such as the cocoons of the primary parasitoid *Cotesia glomerata* (Hymenoptera: Braconidae). *Gelis agilis*, *G. areator* and cocoons of their host, *Co. glomerata* were obtained from existing cultures at the Netherlands Institute of Ecology. *Gelis* sp. were maintained and experiments performed at a relative humidity of 65%, a photoperiod of 16 L : 8 D and a temperature of 25°C. To test larval lipogenic ability, females were first allowed access to 1 to 2 day old host pupae to allow for host-feeding (which is required for oogenesis). After 24 h, females were placed singly in a small Petri dish (3 cm diameter) with access to honey and a single host cocoon for oviposition. Females were observed during 6 h or until oviposition occurred. Two days after oviposition, host cocoon casings were carefully dissected to determine the presence of *Gelis* larvae, which were then separated from the host. An isotope (*n*=5 for *G. agilis* and *G. areator*) or control solution (*n*=5 for *G. agilis* and *n*=3 for *G. areator*) was then randomly applied to each larva until complete absorption, similar to the procedures described for *D. melanogaster*. Larvae were then placed back onto their host to continue development, until they were frozen at −18°C at 6 days of age until further processing. To test adult lipogenic ability, the same procedure was followed as described for *D. melanogaster* (with *n*=15 and *n*=8 each for treatment and control groups of *G. agilis* and *G. areator*, respectively).

### 2.2. Lipid extraction and sample preparation for gas chromatography/mass spectrometry

Insects were homogenized in 1.5 ml microcentrifuge tubes using a plastic pestle, after which 900 μl of chloroform : methanol (2 : 1) was added. Tubes were then shaken at 200 r.p.m. for 15 min and centrifuged at 6000 *g* for 4 min. All solvent was then transferred into a 1 ml microreaction vessel (Supelco, Sigma-Aldrich); 300 μl of 0.9% NaCL solution was added to the solvent and the mixture was vortexed for 30 s. Samples were left at room temperature during 5 min for phases to separate, after which the aqueous upper layer was discarded. Prior to lipid methanolysis (resulting in fatty acid (FA) methylesters), 10 μl of an internal standard (heptadecanoic acid, 100 μg ml^−1^) was added to each sample. The organic phase was then dried down under a nitrogen stream until complete evaporation and 100 μl of methanolic HCl (Supelco, Sigma-Aldrich) was added. The vials were thoroughly capped and then placed in an oven at 65°C. After 30 min, the vials were removed and allowed to cool down before 200 μl of pentane was added. Saturated sodium bicarbonate solution (200 μl) was then added to neutralize the solution, followed by another 200 μl of pentane. Samples were then vortexed for 30 s, after which the top (pentane) layer was transferred into a 0.5 ml Eppendorf tube and centrifuged at 6000 g for 8 min, after which 200 μl was transferred to a GC vial with a 200 μl insert. The vials were stored at −80°C until GC/MS analyses. Prior to GC/MS analyses, the samples were allowed to thaw to room temperature, after which they were vortexed for 2 min at maximum speed before being added to the autosampler tray.

### 2.3. Gas chromatography/mass spectrometry analyses

Analyses were performed on a 7890 GC/7000B triple quadrupole MS system (Agilent Technologies). Sample injection (1 μl, on column) was performed with the injector temperature set to track the GC oven. The carrier gas was He with a constant flow of 35 cm min^−1^. The 30 m×0.25 mm internal diameter, 0.25 μ*m* film thickness HP-5MS column was held at a starting temperature of 30°C for 1 min and then temperature was programmed 15°C min^−1^ to 150°C, followed by 5°C min^−1^ to 280°C and held at that temperature for 5 min. The MS transfer line was set to 260°C. The inert ion source was set to 220°C and tuned to the highest possible resolution (half-height peak width of ±0.3 mass units). To further maximize sensitivity and accuracy, MS data were collected over specified time ranges for each FA methylester by selected ion monitoring of the non-isotope M+ ions and the four nearest isotopic ions (M+1, M+2, M+3 and M+4). A dwell time of 30 ms was used for all mass to charge ratios (*m*/*z*). For each methylester of interest, the following *m*/*z* were used: *m*/*z* 214.2–218.2 for lauric acid (C12 : 0), *m*/*z* 242.2–246.2 for myristic acid (C14 : 0), *m*/*z* 268.2–272.2 for palmitoleic acid (C16 : 1), *m*/*z* 270.2–274.2 for palmitic acid (C16 : 0), *m*/*z* 284.2–288.2 for the internal standard heptadecanoic acid (C17 : 0), *m*/*z* 296.2–300.2 for oleic acid (C18 : 1) and *m*/*z* 298.2–302.2 for stearic acid (C18 : 0).

The GC/MS chromatograms were analysed using MassHunter qualitative analysis software (Agilent Technologies) as total ion current collections where, for each methyl ester, the intensities of the four collected ions (*m*/*z*) were averaged over the total peak. This eliminated errors created by a slightly shifting retention time with added deuterium, resulting in a peak broadening for compounds with a high degree of deuterium incorporation. Abundance of M+1 to M+4 ions was then expressed as a percentage of the parent ion.

### 2.4. Statistical analyses

Deuterium incorporation and error in detection varies by ion (i.e. the probability of finding M+4 ions is lower than M+1 ions and longer chain length FAs have a higher probability of incorporation). Linear mixed-effects models were therefore performed using ion as a random effect to model deuterium incorporation. Models were designed such that species (*E. vuilleti*, *G. agilis*, *G. areator* or *D. melanogaster*), treatment (water or deuterium), and chain length (C12 : 0 + C14 : 0; C16 : 0 + C16 : 1; C18 : 0 + C18 : 1 and the internal standard C17 : 0) and their interactions were incorporated as fixed factors, while ion type (M+1 to M+4) was treated as a random factor with varying slopes and intercepts for chain length and treatment. The best-fit model was chosen based on Bayesian information criterion scores and *χ*^2^ tests on log-likelihood values. Additionally, models were examined to determine adherence to assumptions of residual normality. After investigation with Shapiro–Wilk’s test and visual inspection with quantile–quantile plots, a log transformation was deemed necessary. Post hoc comparisons with Dunnett’s test were used to compare incorporation in deuterium and control treatments. Levels of incorporation for different chain lengths in adults were compared using Tukey’s honest significant difference test.

Data were compiled in Microsoft Excel then analysed in the R (v. 3.2.3) [27] computing environment with the help of the RStudio (v. 0.99.891) development environment [28]. The following packages facilitated data management, analysis and visualization: *dplyr* [29] and *tidyr* [30] for data management, *xlsx* [31] for linking R and Microsoft Excel, *car* [32] for regression analytics, *lme*4 [33] for modelling with linear mixed-effects models, *piecewiseSEM* [34] for *R*^2^ parameters for mixed-effects models, and *lsmeans* [35] for post hoc comparisons.

## 3. Results

### 3.1. Adults

**Figure 1.**
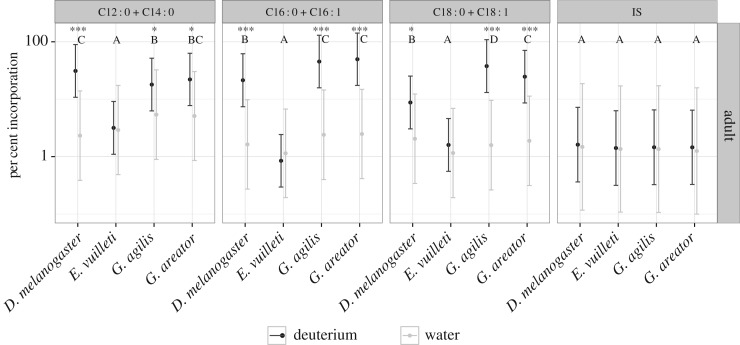
Deuterium incorporation into shorter chain fatty acids (C12 : 0 + C14 : 0), palmitate and palmitoleate (C16 : 0 + C16 : 1) and longer chain fatty acids (C18 : 0 + C18 : 1) for adults. IS refers to the internal standard (C17 : 0) that was added as a control. Asterisks indicate significant differences in incorporation between adults receiving deuterium treatment and those receiving water controls. Points and error bars denote log-transformed adjusted mean per cent incorporation and 95% CIs, respectively. Letters not shared within chain length type indicate significant differences in rates of incorporation between species (*α*=0.05).

After feeding on sugar–water with or without the stable isotope deuterium, incorporation was traced into the FA fraction of all species. Significant main effects were observed for species, treatment and FA chain length, as well as their interactions (figure 1 and table 1). No incorporation of deuterium was observed in the parasitoid *E. vuilleti* (*t*_8.87_=0.13, *p*=0.90), whereas the vinegar fly *D. melanogaster* (*t*_8.15_=5.99, *p*=0.0003), and the parasitoids *G. agilis* (*t*_8.04_=6.60, *p*=0.0002) and *G. areator* (*t*_9.04_=6.31, *p*=0.0001) readily incorporated deuterium in their FA fractions. *Drosophila melanogaster* synthesized the shorter chain FAs (C12 : 0 + C14 : 0) at a higher rate than *G. agilis* and *G. areator* (*t*_2101.32_=20.698, *p*<0.001), whereas the longer chain FAs (C18 : 0 + C18 : 1) were synthesized at higher rates in both *Gelis* species (*p*<0.0001 for all contrasts).

**Table 1.** Analysis of deviance table and model diagnostics from linear mixed-effects models of deuterium incorporation for adults and larvae. (Model statistics are from a log-likelihood ratio comparing the full model with an intercept only model. *χ*^2^, degrees of freedom and *p*-values (*p*) are calculated from Wald-type III tests. *R*^2^ values are marginal (*R*^2^_GLMM(*m*)_) and conditional (*R*^2^_GLMM(*c*)_) *R*^2^ values that describe the proportion of the variance explained by the fixed factors and all factors, respectively [36].)

	adults			larvae		
source	*χ*^2^	d.f.	*p*	*χ*^2^	d.f.	*p*
model	1861.3	31	0.00000	541.25	31	0.00000
intercept	37.0	1	0.00000	4.0	1	0.04657
species (X1)	1211.3	3	0.00000	72.0	3	0.00000
treatment (X2)	14.5	1	0.00014	4.4	1	0.03524
chain length (X3)	26.1	3	0.00001			
X1 × X2	409.3	3	0.00000	29.6	3	0.00000
X1 × X3	751.2	9	0.00000	447.0	24	0.00000
X2 × X3	18.9	3	0.00028			
X1 × X2 × X3	363.1	9	0.00000			
*R*^2^_GLMM(*m*)_	0.400			0.12		
*R*^2^_GLMM(*c*)_	0.872			0.945		

### 3.2. Larvae

**Figure 2.**
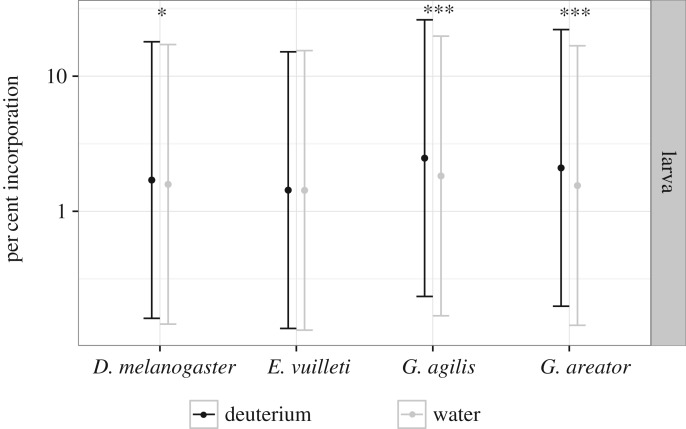
Deuterium incorporation for larvae. Asterisks indicate significant differences in incorporation between larvae receiving the deuterium treatment and those receiving water controls. Points and error bars denote log-transformed adjusted mean per cent incorporation and 95% CIs, respectively.

Isotope tracing following topical application of a water or deuterium solution was performed for larvae of all species. Linear mixed-effects models of larval incorporation showed significant main effects of species and treatment, but no effect of chain length (table 1). Interactions between factors were also significant (table 1). Incorporation was absent in *E. vuilleti* larvae (*t*_30.0_=0.095, *p*=0.925), whereas larvae of *D. melanogaster* (*t*_19.96_=2.11, *p*=0.0475), *G. agilis* (*t*_171.56_=4.936, *p*<0.0001) and *G. areator* (*t*_228.52_=4.495, *p*<0.0001) readily incorporated deuterium in their FA fractions (figure 2) when compared with water-only treatments.

## 4. Discussion

Tracer experiments revealed that larvae of the parasitoid *E. vuilleti* do not synthesize lipids. This lack of lipogenic ability was also found in the adults of this species, in line with previous reports [20,37]. Concurrence in lipogenic strategies was further observed between life stages in the vinegar fly *D. melanogaster* and the parasitoids *G. agilis* and *G. areator*, but unlike *E. vuilleti*, these species were all capable of synthesizing lipids. Although the species that synthesized lipids produced large amounts of palmitate (C16 : 0) as adults, *D. melanogaster* differed from the geline parasitoids by producing shorter chain FAs (C12 : 0+C14 : 0) at a higher rate compared with the longer chain FAs (C18 : 0+C18 : 1) produced by the gelines. A sugar diet was indeed shown to stimulate the production of myristic acid (C14 : 0) in *D. melanogaster* larvae [38] and we found a similar response to sugar feeding in adults. Compared with other labelled FAs, myristic acid (C14 : 0) was previously found to be incorporated at the highest rate in *D. melanogaster* cuticular hydrocarbons [39], chemicals used for interspecies and mate recognition [40]. Stearic acid (C18 : 0) and oleic acid (C18 : 1) are precursors for longer chain FAs, including eicosanoids, which play an important role as signalling molecules [41]. No differences in chain length were found in larvae, potentially owing to the low amount of deuterium available for incorporation in developing larvae (single exposure to approx. 25 nl) compared with that available for adults (ad libitum during 7 days). Despite the lower exposure to isotopes and lower percentage of incorporation, larvae of all species, except *E. vuilleti*, readily incorporated deuterium into their FA fraction.

While the majority of animals readily synthesize lipids, a complete lack of FA synthesis has also been observed in the human pathogenic fungus *Malassezia globosa* [42] and the pathogenic helminth *Schistosoma mansoni* [43] that are both dependent on their hosts for FAs. The sleeping sickness protozoan, *Trypanosoma brucei* was long thought to lack the ability for FA synthesis, but was later found to use a novel FA synthesis pathway based on microsomal elongases [44]. Alternative strategies for the synthesis of shorter chain FAs have further been observed in the aphid *Acyrthosiphon pisum* and *D. melanogaster*. In the former, a thioesterase enzyme cleaves the growing FA chain away from the enzyme fatty acid synthase (FAS) as soon as C14 : 0 is formed [45], whereas in the latter FAS synthesizes C14 : 0 directly [38]. FA synthesis mechanisms in some organisms clearly deviate, sometimes rigorously, from the general metabolic model and modification of lipid synthetic pathways may be more common than currently appreciated. Our data do not, however, suggest that parasitic wasps use alternative pathways for the synthesis of FAs.

Our results revealed that lipid synthesis is either active (*G. agilis* and *G. areator*) or lacking (*E. vuilleti*) throughout the wasps’ life cycle. A previous comparative study already revealed that lipid synthesis was lost in the common ancestor of parasitic wasps as a consequence of the parasitoid lifestyle [22]. This study postulated that lipid synthesis was lost as a consequence of (relaxed) selection on parasitoid larvae that can readily carry over costly lipids from their host (lipid synthesis requires ATP [12]). Lipid synthesis in parasitoid larvae is thus either redundant or too costly to maintain ensuing the loss of this trait. Results on lipid synthesis in adult parasitoids have indeed repeatedly shown consistent patterns: either lipids are synthesized (observed in a few parasitoid species) or they are not (observed in the vast majority of parasitoid species) [22]. Some populations in the parasitoid genus *Leptopilina* were, however, found to vary in their ability for adult lipid synthesis [46]. The majority of adult parasitoids thus show no plasticity in lipogenic ability, but further studies into lipid synthesis of *Leptopilina* populations is necessary to determine whether this variation is owing to plasticity. Our current findings revealed that neither larvae nor adults show plasticity in lipogenic ability and that larval lipid accumulation does not affect lipid synthesis in the adult life stage of the species tested here.

While it is clear that no lipids are produced in either larvae or adults of *E. vuilleti*, we know little about the mechanisms underlying the lack of lipid synthesis in this and other parasitoid species. The lack of lipid synthesis in the human pathogenic fungus *M. globosa* is owing to truncation of a key gene involved in FA synthesis, FAS (*fas*) [42]. Earlier work on the parasitoid *Nasonia vitripennis*, which lacks lipid synthesis, revealed that a functional copy of the *fas* gene is present in its genome without any apparent stop codons in the coding region [21]. *Nasonia vitripennis* does show a deviating transcriptional response to feeding where key genes involved in lipid synthesis are not transcribed, contrary to observations in other animals under the same conditions [47–52]. To fully understand the mechanisms underlying the lack of lipid synthesis in parasitic wasps, we need to dig deeper at the molecular level to uncover which genes and/or regulators are responsible for the lack of lipid synthesis.

## Supplementary Material

Supplementary Data

## Data accessibility

The data supporting this article are available as supplementary information.
